# Depiction of Oral Tumor-Induced Trigeminal Afferent Responses Using Single-Fiber Electrophysiology

**DOI:** 10.1038/s41598-019-39824-9

**Published:** 2019-03-14

**Authors:** Max Grayson, Ashley Furr, Shivani Ruparel

**Affiliations:** Department of Endodontics, University of Texas Health at San Antonio, San Antonio, TX USA

## Abstract

Considerable gap in knowledge exists about the mechanisms by which oral tumors regulate peripheral sensory fibers to produce pain and altered sensations. To address this gap, we used a murine model of oral squamous cell carcinoma (OSCC) of the tongue to investigate changes in response properties of trigeminal afferent neurons. Using this model, we developed an *ex vivo* method for single neuron recordings of the lingual nerve from isolated tongue tissue. Our data demonstrated that the tongue tumor produced increased spontaneous firing of lingual fibers compared to control as well as produced mechanical hypersensitivity and reduced von Frey thresholds of C- and A-slow-high-threshold mechanoreceptors (HTMR) fibers but had no effect on C-LTMR, A-slow-LTMR and A-fast lingual fibers. Mechanically-insensitive fibers were also detected in lingual afferents of the control group, that were significantly decreased in tumor-bearing preparations. Collectively, using single fiber electrophysiology of lingual sensory fibers, we show that human OSCC tumors sensitize peripheral trigeminal nerve terminals, providing a unique opportunity to study mechanisms of oral cancer pain.

## Introduction

Persistent and inadequately treated pain due to head and neck cancer (HNC) causes many patients to seek health care. Indeed, 70–85% HNC patients report pain as their top symptom^1–4^. Most HNC patients require opiates, but rapidly develop tolerance as well as endure long-term adverse effects adding substantially to the emotional burden of having cancer^5^. Clinical studies have shown that HNCs produce pain at the primary site of tumor development such as the tongue, floor of the mouth, larynx, oropharynx, hypopharynx and salivary glands^6,7^. Unlike other cancer types (e.g. breast, prostate and lung), pain is often the first symptom of oral cancer, occurring even when the tumor is quite small in size^6,8–10^. It is therefore likely that oral cancer cells control the activities of surrounding nociceptors at the site of the tumor. Oral cancer cells are heavily innervated with trigeminal afferent terminals. We and others, have reported that tumor cells secrete certain substances such as lipids^11^, nerve growth factor (NGF)^12^, endothelin-1^13^ or ATP^14^ that may stimulate this network of afferent fibers. Additionally, it has been reported that oral cancer patients report intense sharp and aching pain sensations^7^, reminiscent of Aδ and C fiber activation and/or sensitization. Together, these studies strongly implicate a paracrine mechanism for tumor regulation of nociceptor functions.

However, there is a gap in knowledge about the mechanism by which trigeminal fibers are regulated in oral cancer. To address this interaction, we developed an *ex vivo* method for single-fiber recordings of lingual neurons innervating the tongue, similar to the well-recognized method for isolated skin-nerve recordings^15–17^. The current study tests the hypothesis that OSCC tumors sensitize peripheral terminals of trigeminal afferent neurons and identifies the types of fibers regulated by oral tumors. To our knowledge, this is the first study to investigate *ex vivo* single fiber responses of lingual neurons and provides the unique ability to study tumor-nerve interactions in its naturally occurring environment. Single-fiber recordings of the different lingual afferents will offer increased insight into peripheral mechanisms of oral cancer-induced pain as well as serve as a platform to test for analgesic drugs to treat cancer pain.

## Methods

### Cell lines

The human OSCC cell line, HSC2, was purchased from the Health Science Research Resources Bank, Tokyo, Japan. The cell line was cultivated and maintained in Dulbecco’s minimal essential medium (DMEM, Life Technologies, Carlsbad, CA) and supplemented with glutamine, penicillin/streptomycin and 10% fetal bovine serum. The control cell line used was an immortalized human normal oral keratinocyte (NOK) cell line, OKF6-TERT2, which was kindly provided by Dr. Cara Gonzales from University of Texas Health Science Center at San Antonio (UTHSCSA). NOK cells were maintained in keratinocyte serum-free media (Life Technologies, Carlsbad, CA) and supplemented with 25 ug/ml bovine pituitary extract (BPE), 0.2 ng/ml epidermal growth factor (EGF), 0.4 uM calcium chloride and penicillin/streptomycin.

### Animals

The animal protocol was approved by the UTHSCSA IACUC and conforms to the guidelines of International Association for the Study of Pain (IASP). Six to eight-week-old adult inbred Balb/c male athymic nude mice (Charles River, Wilmington, MA, USA) were used for all experiments. Animals were housed for at least 4 days prior to the start of any experiments.

### *In Vivo* Orthotopic Xenograft Model

The mouse tongue cancer pain model was used as described previously^12,14,18–20^. HSC2 cells were harvested in growth media and prepared at a concentration of 3.5 × 10^5^ cells/50uL. Mice were anesthetized using 2% isoflurane inhalation and 50 uL was injected into the ventral tongue over a 30 sec period using a 25-gauge needle. Control groups received 3.5 × 10^5^ NOK cells in the tongue. Following injection, animals were immediately placed in warm bedding and allowed to recover.

### Tongue-lingual nerve dissection

**Figure 1:**
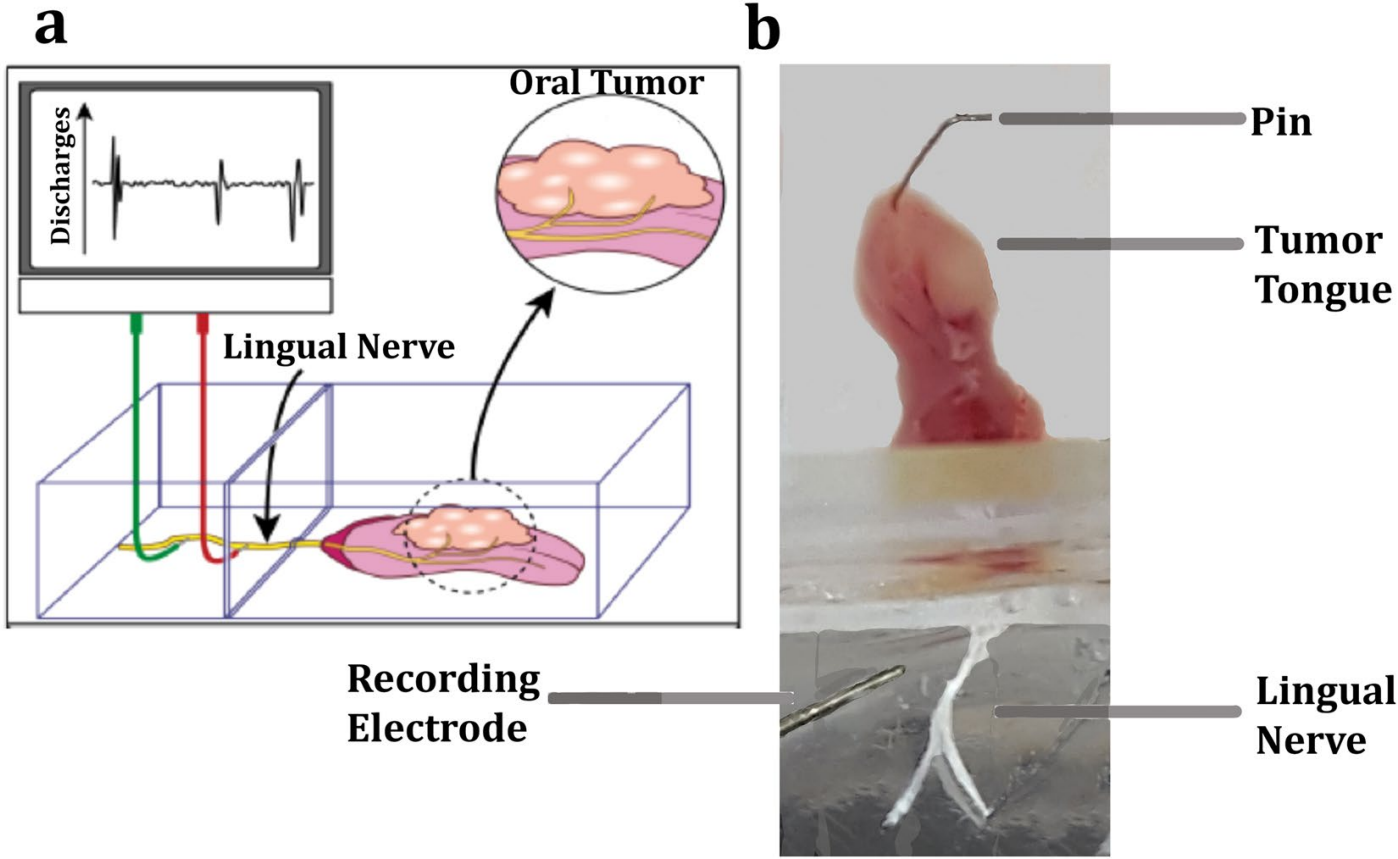
Schematic representation of *ex vivo*-tongue nerve electrophysiology. Oral cancer cells are injected in the anterior of the tongue and, once the tumor has progressed, the lingual nerve and the tongue are dissected. Single fibers of the lingual nerve are isolated, and placed on an electrode, which records discharges upon various noxious stimuli applied onto the tongue. (**b**) Illustration of the *ex vivo* set up showing the tongue of athymic mouse 9 days after inoculation with 3.5 × 10^5^ HSC2 cells. The tongue is pinned down in a chamber containing buffer and the lingual nerve is passed through the second chamber containing a lower layer of buffer and a top layer of mineral oil. The nerve is desheathed, teased into single fibers and connected to the recording electrode to measure discharges upon application of stimuli on the tongue using a CED 1401 and Spike 2 software.

All animals were used at day-9 post cell inoculation. Mice were anesthetized using isoflurane inhalation followed by decapitation. A midline incision was made in the skin from the lower lip into the neck followed by exposure of the tongue by dissecting the digastric muscle located immediately beneath the connective tissue layer. The mandible was cut into half at the incisors to allow for exposure of the mylohyoid muscle which was then dissected to further open the jaw bilaterally. The mandible was then pinned down bilaterally. The tissue around the tongue and the lingual nerve was carefully cut through to expose the lingual nerves bilaterally until the foramen ovale, the exit point of the V3 division of the trigeminal nerve from the skull. Both left and right nerves were cut, and a suture was tied around the free ending. A midline incision was made on the ventral side of the tongue and the preparation was transferred to the recording chamber and the tongue was opened up from the midline and pinned down along its periphery on a silicone layer (Sylgard 184, Dow Corning Corporation, Midland, MI, USA) in the recording chamber (Fig. 1a,b).

### Single-Fiber Electrophysiology

The preparation was placed in a perfusion chamber that was continuously superfused with physiological buffer (123 mM NaCl, 9.5 mM Na-gluconate, 5.5 mM glucose, 7.5 mM Sucrose, 10 mM HEPES, 3.5 mM KCl, 2 mM CaCl_2_, 0.7 mM MgSO_4_, 1.7 mM NaH_2_PO_4_, pH 7.4) and saturated with a mixture of 95% oxygen and 5% carbon dioxide. The temperature of the bath solution was maintained at 32 C. Using the sutures, the nerve was drawn to a recording chamber containing buffer at the bottom and mineral oil as the top layer. The nerve was then de-sheathed and progressively smaller filaments were teased out with sharpened forceps to allow single fiber recordings to be made using extracellular silver-wire recording electrodes (catalog # AGW1010, World Precision Instruments, Sarasota, Florida, USA). Because the lingual nerve is only ~1 cm long, in order to achieve sufficient length to loop the nerve bundle around the electrode, we used a recording electrode with a smaller diameter of 0.25 mm. Neural activity was amplified (DAM50, Harvard Apparatus, Holliston, MA) and filtered. Amplified signals were led to a digital oscilloscope (Tektronix TBS 1072B-EDU, Fotronic Corporation, Melrose, Massachusetts, USA) and an audio monitor (AM 10 Audio Monitor, Grass Technologies, Warwick, Rhode Island, USA) and fed into PC computer via a data acquisition system (CED1401/Spike 2, Cambridge Electronic Design Ltd., Cambridge, UK). For data inclusion, single fiber recordings were verified by a marking protocol (or collision method) as described previously^15,21^. Action potentials collected on a computer were analyzed off-line with a template-matching function of Spike 2 software (Cambridge Electronic Design Ltd.). Receptive fields (RF) were identified by electrical stimulation using a low-impedance (50–100 kOhm) 15mm-long tungsten electrode (FHC, Inc., Bowdoin, Maine, USA) and current stimulus of 0.2–6.0 mA, generated by a stimulus isolator (Model A365, World Precision Instruments, Sarasota, Florida, USA). Only units with a clearly distinguished signal to noise ratio (greater than 2:1) were further studied. Upon identification, sensitivity to mechanical stimulation of the fibers was determined by probing the tongue with a blunt glass rod. Fibers unresponsive to the glass rod were considered mechanically insensitive afferent (MIA) fibers. The mechanical threshold of a mechanically-sensitive fiber was determined using calibrated von Frey filaments (0.44–200 mN). Next, the conduction velocity (CV) of each fiber was determined by electrical stimulation of the receptive field using a low-impedance (50–100 kOhm) 15mm-long tungsten microelectrode (FHC, Inc., Bowdoin, Maine, USA). The distance between the receptive field and the recording electrode was divided by the latency of the action potential to determine CV. Afferent fibers conducting slower than 1 m/s were classified as C-fibers; those conducting between 1 and 3.0 m/s as A-slow, and those conducting faster than 3 m/s as A- fast fibers. To determine quantitative mechanical sensitivity, a feedback-controlled constant-force mechanical stimulator (Series 300B Dual Mode Servo System, Aurora Scientific, Aurora, Ontario, Canada) was used. A flat-ended cylindrical metal probe (tip diameter of 0.7 mm) attached to the tip of the stimulator arm was placed at the most sensitive spot of the receptive field with no force generated. After a 2-min baseline recording, a computer-controlled ascending series of square force stimuli was applied at 60-s intervals with force stimuli of 10–200 mN. Fibers were further classified according to their responses to sustained mechanical forces into low-threshold mechanoreceptors (LTMR) and high threshold mechanoreceptors (HTMR) or nociceptors, and adaptation responses to sustained suprathreshold mechanical stimuli (Slowly Adapting fibers (SA) versus Rapidly Adapting (RA) fibers. Fibers were classified as HTMR or LTMR based on their firing patterns upon the step-and hold automated mechanical ramp as reported previously^22^. Fibers that produced increase in number of impulses with increasing force were classified as HTMR whereas those that responded with higher impulses at lower forces and then either plateaued or decreased in impulses at higher forces were considered LTMR fibers. Because mechanical thresholds of lingual fibers can be different from cutaneous fibers, we did not use von Frey thresholds to determine HTMR versus LTMR. For determination of spontaneous activity, fibers that exhibited sustained discharges (2–3 bouts of activity per 20 secs for burst firing or at a frequency of at least 0.2 Hz for continuous firing) immediately after placing the filament on the recording electrode, were considered spontaneously active fibers. Rates of discharge of ongoing activity was calculated as number of impulses in one second (Hz) and peak discharge was calculated as the maximal rate of discharge based on the shortest time between two action potentials.

### Statistics

Data are presented as mean ± SEM. Statistical analyses included Mann-Whitney U test to compare peak discharge rate of spontaneously active fibers with p < 0.05 as significant. Fisher’s exact test was used to compare differences in occurrence of spontaneous fibers in normal and tumor groups. Unpaired t-test at p < 0.05 significance, was used to compare differences in conduction velocities and von Frey Thresholds. Two-way ANOVA with Bonferroni post-hoc test was used to determine statistical significance of mechanically-stimulated impulses between control and tumor-bearing preparations.

## Results

**Figure 2:**
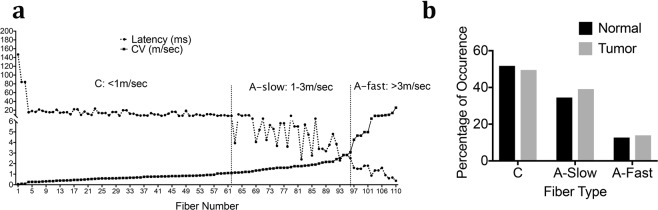
CV Distribution of mouse lingual afferents and occurrence. (**a**) Plot of CV against latency of 110 single fibers from normal mouse tongue. CV of C-fibers was <1 m/sec, A-slow fibers was <3 m/sec and A-fast fibers was >3 m/sec. (**b**) Percentage of occurrence of C, A-slow and A-fast fibers from normal and HSC-2 tongue tumor-bearing preparations in mice.

Because this is the first study evaluating *ex vivo* mouse lingual afferents, we first determined the range of conduction velocities for C and A fibers. CVs of 110 fibers from normal tongue preparation were recorded and plotted against latency as shown in Fig. 2a. Based on the distribution, we determined that CVs of C-fibers to be <1 m/sec, and the A fibers were divided into A-slow <3 m/s and A-fast >3 m/s (Fig. 2a). These cut-offs of A-slow and A-fast fibers are similar to previous reports of recordings from trigeminal dural afferents in rats where the cut offs of A-fast fibers were >5 m/sec^23^. The A-fast group therefore includes neurons in the A**β** and upper range of A**δ** fibers. As seen in Fig. 2a, the number of fibers encountered in the accepted A**β** range (>8–10 m/sec) were very few. We also observed that the percentage of occurrence of each of the fiber type was C > A-slow > A-fast and this did not change in tumor-bearing preparations (Fig. 2b). We then recorded a total of another 93 fibers from normal tongue-nerve preparations and 135 fibers from tumor bearing preparation to evaluate spontaneous activity and mechanical responses in both groups.

### Spontaneous Activity in Fibers from Control and Tumor-bearing Preparations

**Figure 3:**
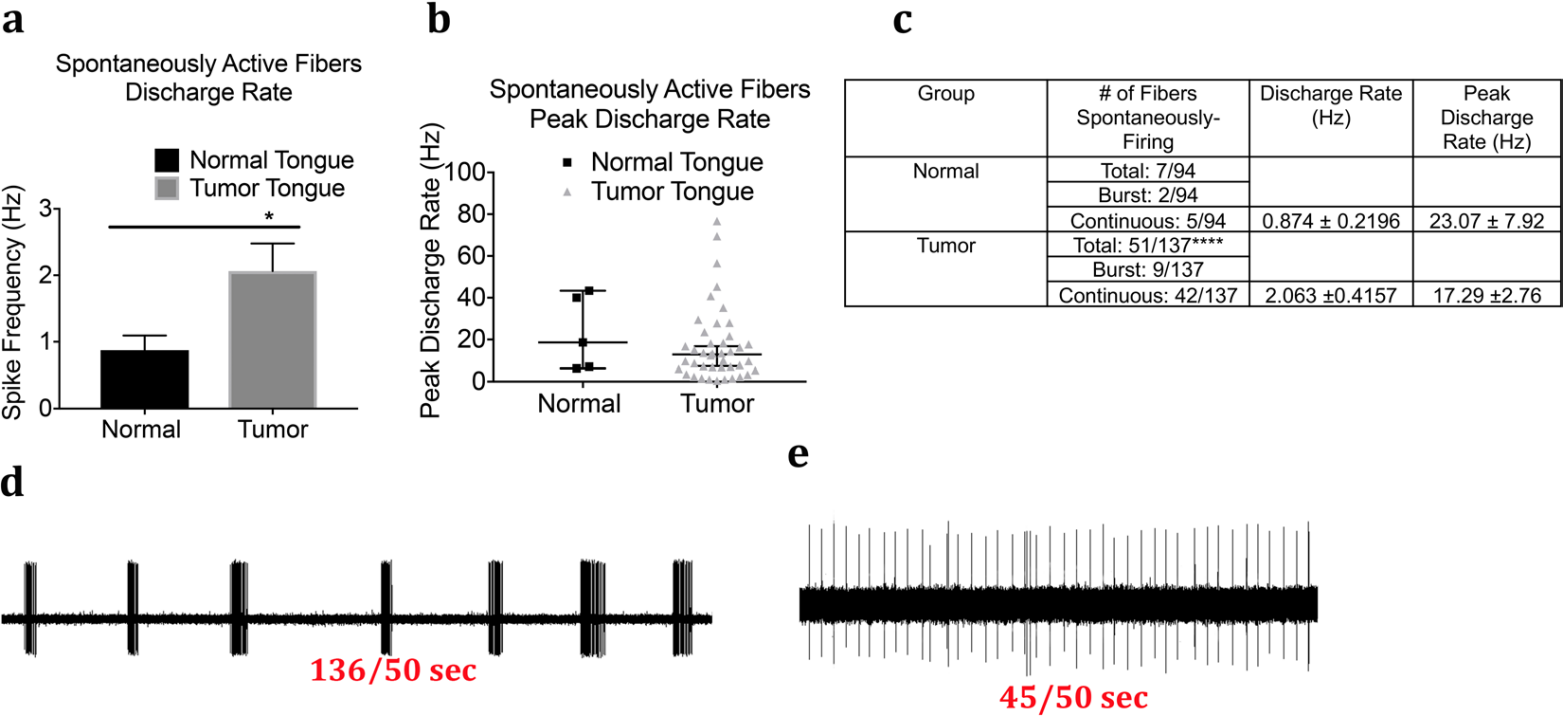
Effect of tongue tumor on spontaneous activity of lingual afferents. Normal and tumor-bearing preparations at day 9 post OSCC inoculation were evaluated for spontaneous activity of single fibers. (**a**) Discharge rate of continuous ongoing activity of single fibers recorded from normal and tumor-bearing preparations. Discharge rate was calculated as number of impulses per second. (**b**) Peak discharge rate of spontaneously firing fibers from normal and tumor-bearing preparations. Peak Discharge rate was calculated in Hz as the shortest time interval between two action potentials. (**c**) Table summarizing the spontaneously firing fibers in normal and tumor group. Data are presented as mean ± SEM. Significance at p < 0.05. (**d**) Representative trace showing burst firing fiber in the tumor group. (**e**) Representative trace showing continously firing of ongoing activity in the tumor group.

The mean and peak rates of continuous spontaneous discharges were significantly greater in the tumor bearing group as compared to the control group (Fig. 3a–c). In addition to greater discharge rates of afferent fibers in the tumor group, the proportion of fibers exhibiting spontaneous activity was also significantly higher (p < 0.0001); of the fibers recorded, 51 of 135 (37.7%) had spontaneous activity in the tumor group as compared to 7 of 93 (7.5%) in the control group (Fig. 3c). The pattern of discharges included continuous firing as well as intermittent burst firing (Fig. 3d,e) where majority of the fibers recorded were continuously active and a small proportion produced intermittent burst firing (Fig. 3c). Further intermittent burst firing did not show a uniform pattern of bursts each time and therefore discharge rate and peak discharge plotted in Fig. 3a,b were only calculated for continuously active spontaneous fibers. The majority of the spontaneously active fibers were unidentified, however, four fibers in the tumor group were identified out of which one was a C fiber, two A-slow and one A-fast fibers.

### Responses to Mechanical Stimuli in Control and Tumor-bearing Preparations

**Figure 4:**
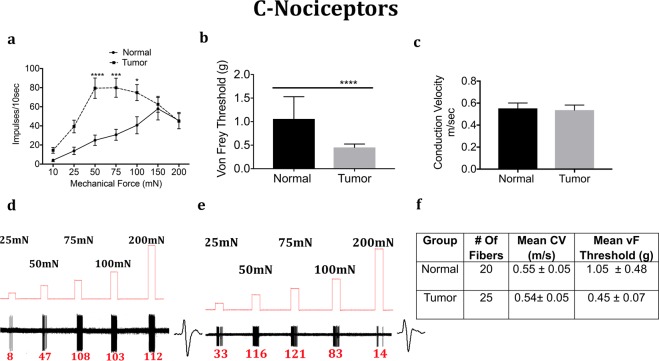
Effect of tongue tumor on mechanical sensitivity of C- nociceptors of lingual afferents. Normal and tumor-bearing preparations at day 9 post HSC2 inoculation were evaluated for mechanical responses of C-nociceptors fibers. (**a**) Impulses of C- fibers from normal and tumor-bearing preparations to sustained mechanical forces. Data are presented as mean ± SEM. Data are analyzed with one-way ANOVA with Bonferroni post hoc test at p < 0.05. n = 13 fibers in normal group and n = 17 fibers in tumor group. (**b**) von Frey Thresholds of C fibers from normal and tumor group. Data are presented as mean ± SEM. Data are analyzed with Mann Whitney U test at p < 0.05. (**c**) Conduction velocity of C fibers from normal and tumor group. Data are presented as mean ± SEM. Data are analyzed with unpaired t-test at p < 0.05. (**d**,**e**) Representative traces for normal and tumor groups showing C-fiber responses upon sustained mechanical forces. Numbers in red indicate number of impulses per force applied. (**f**) Table lists the number of C-nociceptors fibers tested, mean conduction velocities (CV) and mean von Frey thresholds for both groups.

**Figure 5:**
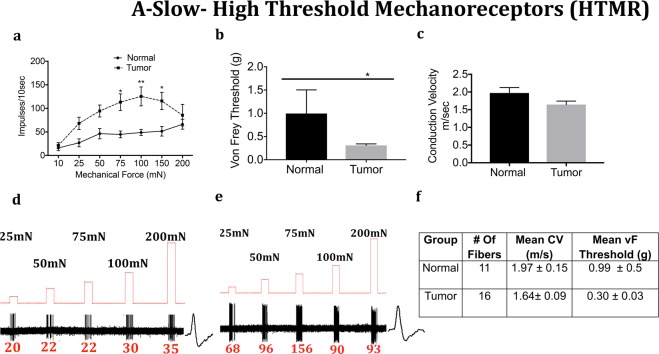
Effect of tongue tumor on High Threshold A-Slow- Mechanoreceptors of lingual afferents. Normal and tumor-bearing preparations at day 9 post HSC2 inoculation were evaluated for mechanical responses of A-slow -HTMR fibers. (**a**) Impulses of A-slow-HTMR fibers from normal and tumor-bearing preparations to sustained mechanical forces. Data are presented as mean ± SEM. Data are analyzed with one-way ANOVA with Bonferroni post hoc test at p < 0.05. n = 13 fibers in normal group and n = 17 fibers in tumor group. (**b)** von Frey Thresholds of A-slow -HTMR fibers from normal and tumor group. Data are presented as mean ± SEM. Data are analyzed with Mann Whitney U test at p < 0.05. (**c**) Conduction velocity of A-slow -HTMR fibers from normal and tumor group. Data are presented as mean ± SEM. Data are analyzed with unpaired t-test at p < 0.05. (**d**,**e**) Representative traces for normal and tumor groups showing A**- slow**-HTMR fiber responses upon sustained mechanical forces. Numbers in red indicate number of impulses per force applied. (**f**) Table lists the number of A- slow -HTMR fibers tested, mean conduction velocities (CV) and mean von Frey thresholds for both groups.

**Figure 6:**
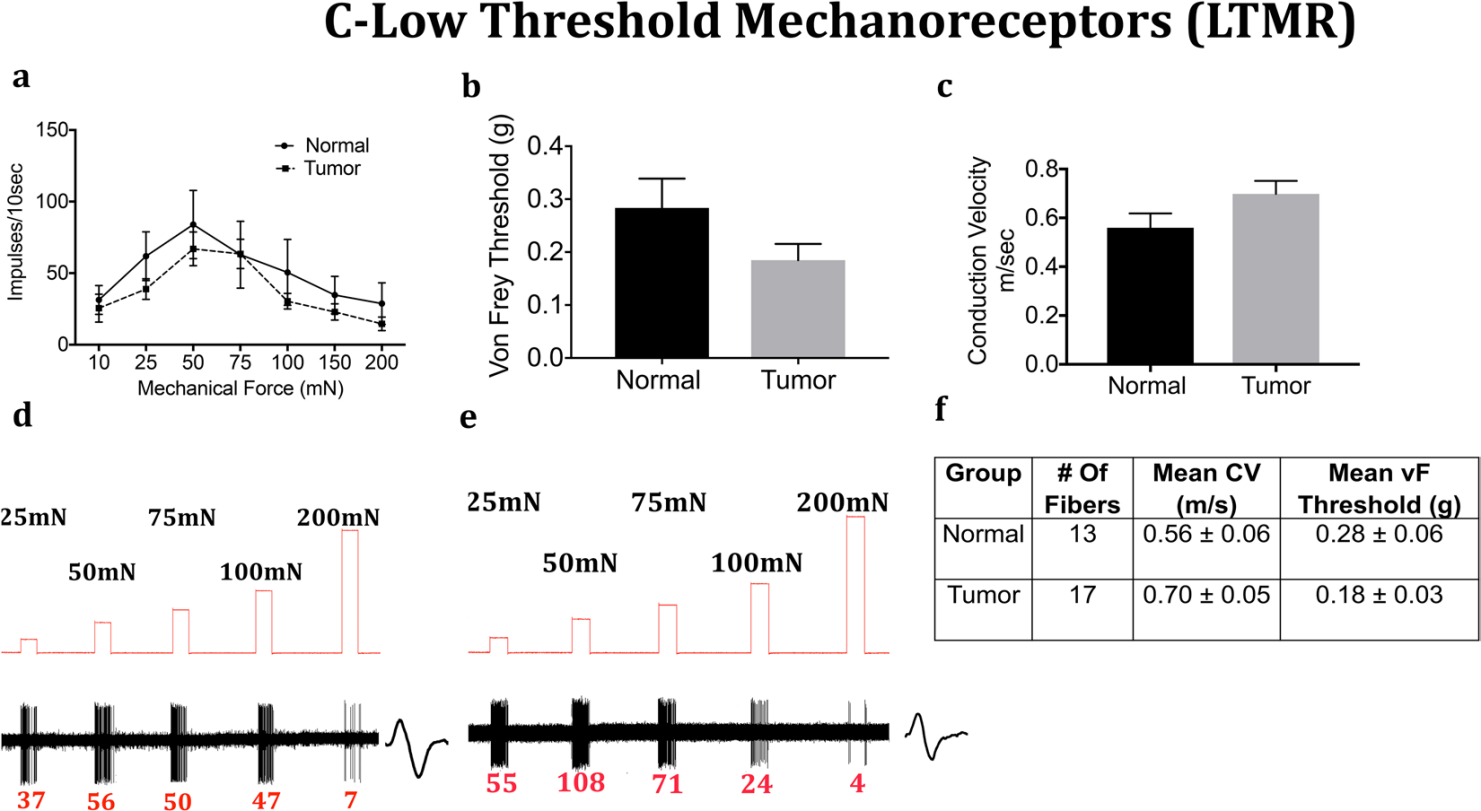
Effect of tongue tumor on Low Threshold C- Mechanoreceptors of lingual afferents. Normal and tumor-bearing preparations at day 9 post HSC2 inoculation were evaluated for mechanical responses of C-LTMR fibers. (**a**) Impulses of C-LTMR fibers from normal and tumor-bearing preparations to sustained mechanical forces. Data are presented as mean ± SEM. Data are analyzed with one-way ANOVA with Bonferroni post hoc test at p < 0.05. n = 13 fibers in normal group and n = 17 fibers in tumor group. (**b**) von Frey Thresholds of C-LTMR fibers from normal and tumor group. Data are presented as mean ± SEM. Data are analyzed with Mann Whitney U test at p < 0.05. (**c**) Conduction velocity of C-LTMR fibers from normal and tumor group. Data are presented as mean ± SEM. Data are analyzed with unpaired t-test at p < 0.05. (**d**,**e**) Representative traces for normal and tumor groups showing C-LTMR fiber responses upon sustained mechanical forces. Numbers in red indicate number of impulses per force applied. (**f**) Table lists the number of C-LTMR fibers tested, mean conduction velocities (CV) and mean von Frey thresholds for both groups.

**Figure 7:**
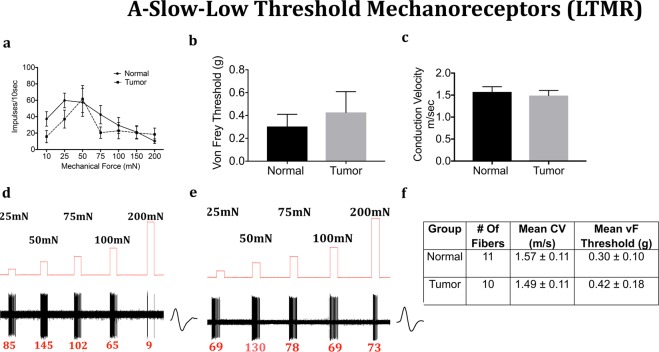
Effect of tongue tumor on Low Threshold A-Slow- Mechanoreceptors of lingual afferents. Normal and tumor-bearing preparations at day 9 post HSC2 inoculation were evaluated for mechanical responses of A-slow -LTMR fibers. (**a**) Impulses of A-slow -LTMR fibers from normal and tumor-bearing preparations to sustained mechanical forces. Data are presented as mean ± SEM. Data are analyzed with one-way ANOVA with Bonferroni post hoc test at p < 0.05. n = 13 fibers in normal group and n = 17 fibers in tumor group. (**b**) von Frey Thresholds of A-slow -LTMR fibers from normal and tumor group. Data are presented as mean ± SEM. Data are analyzed with Mann Whitney U test at p < 0.05. (**c**) Conduction velocity of A-slow -LTMR fibers from normal and tumor group. Data are presented as mean ± SEM. Data are analyzed with unpaired t-test at p < 0.05. (**d**,**e**) Representative traces for normal and tumor groups showing A-slow -LTMR fiber responses upon sustained mechanical forces. Numbers in red indicate number of impulses per force applied. (**f**) Table lists the number of A-slow -LTMR fibers tested, mean conduction velocities (CV) and mean von Frey thresholds for both groups.

**Figure 8:**
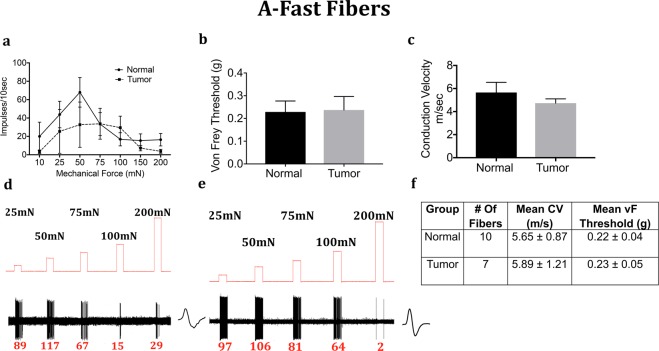
Effect of tongue tumor on mechanical sensitivity of A-Fast fibers of lingual afferents. Normal and tumor-bearing preparations at day 9 post HSC2 inoculation were evaluated for mechanical responses of A-fast fibers. (**a**) Impulses of A-fast fibers from normal and tumor-bearing preparations to sustained mechanical forces. Data are presented as mean ± SEM. Data are analyzed with one-way ANOVA with Bonferroni post hoc test at p < 0.05. n = 13 fibers in normal group and n = 17 fibers in tumor group. (**b**) von Frey Thresholds of A-fast fibers from normal and tumor group. Data are presented as mean ± SEM. Data are analyzed with Mann Whitney U test at p < 0.05. (**c**) Conduction velocity of A-fast fibers from normal and tumor group. Data are presented as mean ± SEM. Data are analyzed with unpaired t-test at p < 0.05. (**d**,**e**) Representative traces for normal and tumor groups showing A-fast fiber responses upon sustained mechanical forces. Numbers in red indicate number of impulses per force applied. (**f**) Table lists the number of A-fast fibers tested, mean conduction velocities (CV) and mean von Frey thresholds for both groups.

We next determined whether the mechanical response properties of lingual afferents are regulated after tumor growth in mice. Fibers were tested for von Frey threshold as well as number of discharges upon automated step-and-hold ramps of mechanical force ranging from 10–200 mN. Each fiber was identified to be C, A-slow or A-fast and further classified into LTMR or HTMR as described in the methods. C-fibers innervating the tongue tumor demonstrated significantly greater responses to mechanical ramps (Fig. 4a,d,e) and lower von Frey thresholds (Fig. 4b) as compared to C-fibers innervating control tongue tissue. In contrast, there was no difference in conduction velocity (Fig. 4c). Similar to the C-HTMRs, the A-slow-HTMRs innervating the tongue tumor demonstrated significantly greater responses to mechanical ramps (Fig. 5a,d,e) and lower von Frey thresholds (Fig. 5b) as compared to A-slow- HTMRs innervating control tongue tissue. Again, there was no difference in conduction velocity (Fig. 5c). The effect of the OSCC tumors on afferent responsiveness was selectively restricted to the HTMR subpopulations, as it was not observed in the LTMR subpopulations characterized as C **(**Fig. 6), A-slow (Fig. 7) or A-fast (Fig. 8) fibers.

Another feature observed in lingual sensory fibers was the smaller occurrence of rapidly adapting fibers compared to slowly adapting mechano-fibers. Of all the mechanically-sensitive fibers recorded, we only found 4 fibers that were rapidly adapting whereas the majority of the fibers were slowly adapting fibers (data not shown).

**Figure 9:**
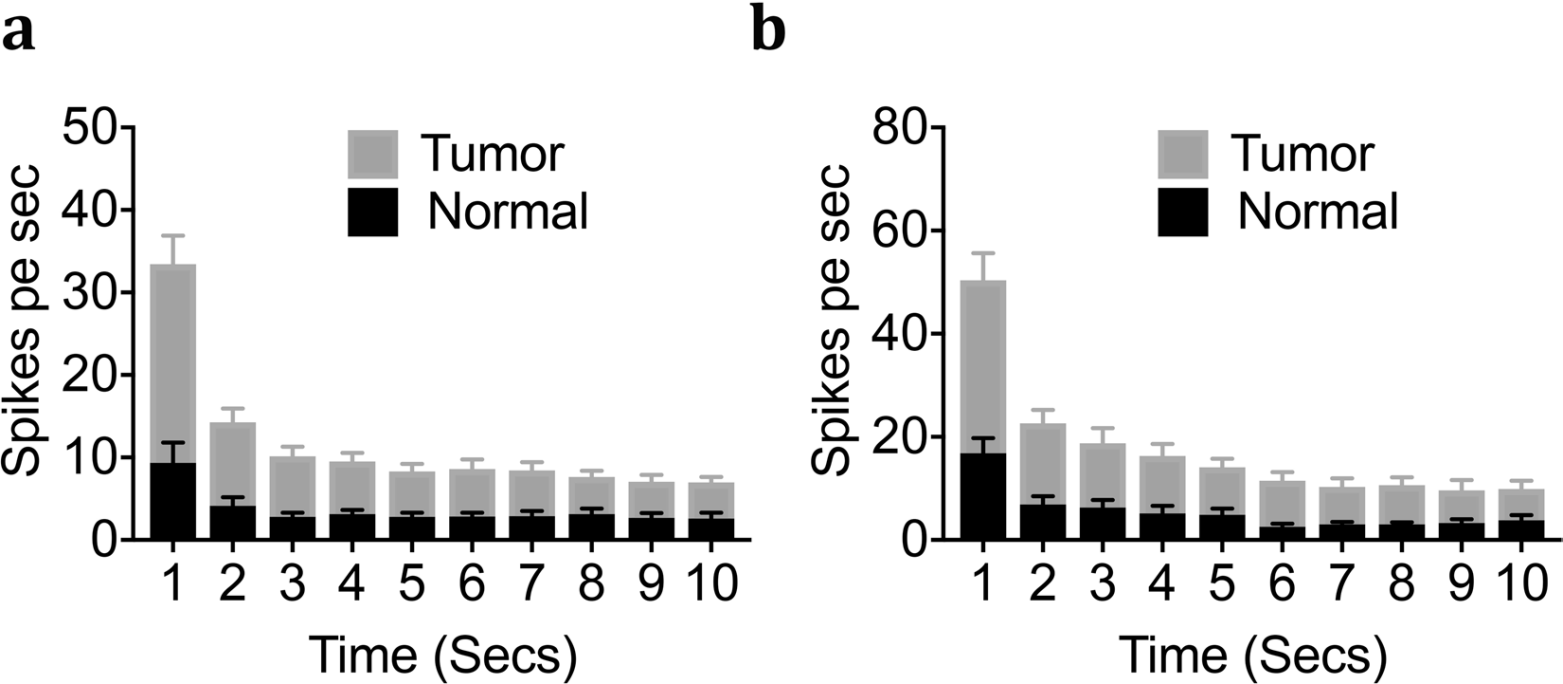
Effect of tongue tumor on adaptation Pattern of C and A-Slow nociceptors. NOK or HSC2-injected tongue-nerve preparations were evaluated for adaptation pattern at a sustained mechanical force. Impulses per second during a 10 second sustained mechanical stimuli of (**a**) C nociceptors at 75 mN and (**b**) A-slow-HTMR at 100 mN. Data are presented as mean ± SEM.

We also examined adaptation rates to sustained force for each fiber type of slowly adapting fibers. Adaptation properties of C- and A-slow-HTMR afferents were tested at a 10-second sustained high-intensity force of 75 mN and 100 mN respectively. As seen in Fig. 9a,b, both fiber types had similar adaptation rates and produced sustained firing throughout the duration of force applied, in tumor-bearing and control preparations.

In addition to mechanically-sensitive fibers, we also were able to detect mechanically-insensitive fibers in lingual afferents. Interestingly, the number of MIA fibers detected in the control group was higher compared to tumor-bearing group (Table 1). Of the MIA fibers recorded, majority of the fibers were identified to be C-fibers and a few to be A-slow-fibers. These data indicate a potential conversion of MI fibers to mechanically-sensitive fibers upon tumor growth.

**Table 1:** Mechanically-insensitive fibers in normal and tumor-bearing lingual afferents.

Group	Fiber Type	^#^Of Fibers	Mean CV(m/s)
Normal	A-fast	0	—
	A-slow	5	1.3 ± 0.11
	C	16	0.5 ± 0.07
Tumor	A-fast	0	—
	A-slow	3	1.67 ± 0.278
	C	6	0.62 ± 0.06

## Discussion

Pain is the top ranked symptom in head and neck cancer patients and, unlike other cancers, is produced at the primary site of tumor development suggesting that tumor cells regulate the activities of peripheral terminals of trigeminal primary afferents^6,8–10,24–26^. We and others have reported that oral tumor cells can regulate the peripheral pain pathway by releasing several substances that activate/sensitize peripheral sensory afferents^11–14^. However, detailed characterization of the regulation of the different types of sensory nerve terminals in oral cancer is limited. To address this gap, the current study employed a previously reported orthotopic tongue cancer pain model in mice^20^, that mimics patient reported symptoms with an *ex vivo* single-fiber recording method to identify types of sensory afferents and characterize the firing responses with and without tumor growth. The method allows the investigation of peripheral mechanisms by which oral tumors induce pain in its naturally occurring tissue environment. The HSC-2 human OSCC cell line or NOK cells (as a control) were injected into the tongues of athymic mice and at day 9 post-inoculation, the tongue with the associated lingual nerves were dissected and used to study firing discharges of single fibers upon stimulation of the tongue (Fig. 1a,b). Because oral cancer patients complain of ongoing pain as well as function-related pain such as pain while chewing and swallowing^6,7,27^, we determined whether the OSCC tongue tumor in mice produced spontaneous discharges as well as mechanical hypersensitivity of lingual afferents. Our data demonstrated that the occurrence of spontaneously active fibers was significantly increased in tumor-bearing preparations compared to control preparations. (Fig. 3c). Moreover, the discharge rate of continuously firing fibers was significantly higher in tumor-bearing preparations compared to the control preparations (Fig. 3a). Several reports have implicated spontaneous activity of sensory neurons due to central sensitization^28,29^. However, our data using an *ex vivo* method suggest that oral tumor induced spontaneous activity may be partly due to tumor cell regulation of peripheral afferents. These findings are similar to previously reported spontaneous activity of tibial nerve fibers in a mouse incision model^30^ as well as of sciatic nerve fibers in a murine bone cancer pain model^31^. The peripheral mechanism(s) by which tumor cells induce spontaneous activity is yet to be determined and may differ among various tumor types. Several reported substances such as lipids^11^ and ATP^14^, released by tumor cells can continuously activate nociceptors whereas other reported mediators like NGF^12^ and enothelin-1^32^ can produce a significant afferent barrage resulting in ongoing depolarization of lingual afferents. These hypotheses can be explored in the future using the employed single-fiber electrophysiology.

We also determined the range of CVs of lingual afferents by plotting a distribution of latency against CVs of 110 fibers. We note that there are several limitations of using CVs as a basis of identifying fibers as reported previously^23,33^ and accurate identification of fibers can be compromising especially within the A fiber group. Therefore, based on our observed latency versus CV distribution and previously reported CV cutoffs for trigeminal afferents^23^ we classified fibers to be C <1 m/sec, A-slow <3 m/sec and A-fast >3 m/sec. Among the A-fast fibers, lingual afferents falling under the CV range of accepted A**β** group (>8–10 m/sec), were found to be very few. A possible reason for this could be that the axon diameter and myelination of lingual afferents may be significantly different from other reported sensory nerves to where CVs of A**β** fibers may be considerably lower. Additionally, Levy *et al*.^23^ have discussed probabilities of underestimation of CVs: (1) if the fiber within the nerve takes a more circuitous route compared to a direct route; and (2) if CVs of axonal ending slow down after entering into the peripheral tissue. Additional set of extensive characterization such as anatomical determination of axon diameter of lingual sensory neurons, identification of subtype markers using single-cell sequencing as well as use of combined optogenetics and single-fiber electrophysiology, are required to accurately determine the CV cut offs, especially to identify A**β** from the A**δ** group.

Mechanical stimulation of the tongue revealed significant differences in thresholds and firing rates of lingual afferents between control and tumor-bearing groups. Fiber types were identified as C, A-slow and A-fast according to the above-mentioned CV cut offs and were further classified as low or high-threshold mechanoreceptors based on the firing pattern to sustain forces. Our data showed that the OSCC tongue tumor lowered von Frey thresholds and increased discharges to sustained forces of C- and A-slow-HTMRs, whereas response properties of C-LTMR, A-slow -LTMR and A-fast were not altered (Figs 4–8). Additionally, despite increased firing of C and A-slow fibers within the tumor group, these fibers showed increased firing throughout to sustained force and did not adapt any faster (Fig. 9). These data suggest that OSCC cells may regulate specific nociceptors via peripheral mechanisms. As this effect was restricted to HTMR fibers it is likely due to a selective effect, rather than a pan-neuronal mechanism. Recently, we have shown that several channels previously reported to be involved with peripheral mechanical sensitivity such as ASIC1^34,35^, Kv7.2^36^, and Par-2^19^ are upregulated in the V3 region of the TG after tongue tumorigenesis^20^. Investigating the contribution of these channels in OSCC-regulation of specific class of sensory neurons, can significantly address the gaps about mechanisms of mechanical hypersensitivity in oral cancer. Additionally, tumor secreted substances like lipids, ATP^14^, NGF^12^ and serine proteases^37^ have also shown to play a role in mediating mechanosensitivity of lingual afferents in oral cancer.

The finding of no change in conduction velocities after tumor growth indicates that OSCC cells may not alter myelination of sensory afferents at day 9 post-inoculation. Activity-dependent slowing of conduction velocity was not assessed in the study.

We also assessed the presence of mechanically-insensitive afferent fibers in lingual terminals and showed that the detected prevalence of MIA fibers in control preparations was significantly smaller in tumor-bearing preparations (6.6% in tumor vs 22.5% in controls) suggesting that the OSCC tongue tumor may play a critical role in converting MIA fibers into mechanically sensitive fibers. Recently, Prato *et al*., demonstrated that mechanically-insensitive C-Fibers that express the marker CHRNA3 are converted to mechanosensitive neurons in the presence of NGF^38^. Interestingly, CHRNA3 is expressed in lingual neurons^39^ and the majority of MIA fibers detected in our study were C-fibers (Table 1). Moreover, NGF is shown to be overexpressed and released by OSCC tumor contributing to oral cancer pain^12^. These data suggest a potential important regulation of MIA sensory fibers in oral cancer.

In addition to the findings described above, use of tongue-nerve electrophysiology revealed three interesting observations about trigeminal afferent responses. First, C-LTMR fibers were identified in lingual sensory neurons, despite the presence of very low proportion of tyrosine hydroxylase or VGLUT3 expression (both being markers of C-LTMR in dorsal root ganglion (DRG) neurons as reported recently^39–41^. This reflects and emphasizes the differences between DRG neurons and trigeminal ganglia (TG) neurons. While the function of C-LTMR in the skin is shown to be pleasant touch^42^, precise role of C-LTMR in the tongue is unknown. Identification of specific markers for this class of lingual sensory neurons can allow to elucidate its function using the tongue-nerve electrophysiology method. Second, our data may also signal similarities between DRG and TG neurons. Calbindin, a marker of rapidly-adapting neurons^43^ was identified in a very small proportion of lingual neurons^39^. This finding correlated with very few rapidly-adapting fibers that were identified in the tongue-nerve preparations. Third, while A**δ** -LTMR (or D hair) fibers in hairy hindpaw mouse skin are rapidly adapting^44^, the A-slow-LTMR in lingual afferents are slowly adapting fibers. This reflects the tissue-specific differences of sensory afferents, further indicating tissue specific differences in function of sensory fiber subtypes.

Taken together, the current study characterizes mouse lingual afferent properties in response to oral tumor growth at the orthotopic, primary site of tumor development and identifies the sensory neuronal subtypes that may be involved in peripheral mechanisms in oral cancer pain. While lingual nerve recordings have been made *in vivo* in prior reports^45–47^, this is the first study using *ex vivo* single-fiber electrophysiology for lingual afferents permitting the evaluation of peripheral mechanisms of sensory neuron regulation. Moreover, no study has used single-fiber recordings of lingual afferents in an oral cancer pain model that characterizes tumor-nerve interactions *ex vivo*. This is also the first study to demonstrate *ex vivo* single-fiber recordings of trigeminal afferents and can significantly advance the field of oral somatosensation. This method can be employed in future studies aimed at determining the mechanisms for sensitization of primary afferent trigeminal neurons in not only in oral cancer but also in other craniofacial conditions such as oral mucositis, trigeminal neuralgia and burning mouth syndrome.

## References

[1] Chaplin JM, Morton RP (1999). A prospective, longitudinal study of pain in head and neck cancer patients. Head & neck.

[2] Epstein JB, Stewart KH (1993). Radiation therapy and pain in patients with head and neck cancer. European journal of cancer. Part B, Oral oncology.

[3] Keefe FJ, Manuel G, Brantley A, Crisson J (1986). Pain in the head and neck cancer patient: changes over treatment. Head & neck surgery.

[4] Saxena A, Gnanasekaran N, Andley M (1995). An epidemiological study of prevalence of pain in head & neck cancers. The Indian journal of medical research.

[5] Wilson J, Stack C, Hester J (2014). Recent advances in cancer pain management. F1000prime reports.

[6] Cuffari L, T de Siqueira JT, Nemr K, Rapaport A (2006). Pain complaint as the first symptom of oral cancer: a descriptive study. Oral surgery, oral medicine, oral pathology, oral radiology, and endodontics.

[7] Lam DK, Schmidt BL (2011). Orofacial pain onset predicts transition to head and neck cancer. Pain.

[8] Connelly ST, Schmidt BL (2004). Evaluation of pain in patients with oral squamous cell carcinoma. The journal of pain: official journal of the American Pain Society.

[9] Epstein JB, Elad S, Eliav E, Jurevic R, Benoliel R (2007). Orofacial pain in cancer: part II–clinical perspectives and management. Journal of dental research.

[10] Marshall JA, Mahanna GK (1997). Cancer in the differential diagnosis of orofacial pain. Dental clinics of North America.

[11] Ruparel S, Bendele M, Wallace A, Green D (2015). Released lipids regulate transient receptor potential channel (TRP)-dependent oral cancer pain. Mol Pain.

[12] Ye Y (2011). Nerve growth factor links oral cancer progression, pain, and cachexia. Molecular cancer therapeutics.

[13] Schmidt BL (2007). Peripheral endothelin A receptor antagonism attenuates carcinoma-induced pain. European journal of pain.

[14] Ye Y (2014). Adenosine triphosphate drives head and neck cancer pain through P2X2/3 heterotrimers. Acta neuropathologica communications.

[15] Zimmermann K (2009). Phenotyping sensory nerve endings *in vitro* in the mouse. Nature protocols.

[16] Banik RK, Brennan TJ (2008). Sensitization of primary afferents to mechanical and heat stimuli after incision in a novel *in vitro* mouse glabrous skin-nerve preparation. Pain.

[17] Stucky CL, Koltzenburg M (1997). The low-affinity neurotrophin receptor p75 regulates the function but not the selective survival of specific subpopulations of sensory neurons. The Journal of neuroscience: the official journal of the Society for Neuroscience.

[18] Dolan JC, Lam DK, Achdjian SH, Schmidt BL (2010). The dolognawmeter: a novel instrument and assay to quantify nociception in rodent models of orofacial pain. Journal of neuroscience methods.

[19] Lam DK, Dang D, Zhang J, Dolan JC, Schmidt BL (2012). Novel animal models of acute and chronic cancer pain: a pivotal role for PAR2. The Journal of neuroscience: the official journal of the Society for Neuroscience.

[20] Chodroff, L., Bendele, M., Valenzuela, V., Henry, M. & Ruparel, S. EXPRESS: BDNF Signaling Contributes to Oral Cancer Pain in a Preclinical Orthotopic Rodent Model. *Mol Pain* **12**, 10.1177/1744806916666841 (2016). 10.1177/1744806916666841 PMC5015823 27590070

[21] Kress M, Koltzenburg M, Reeh PW, Handwerker HO (1992). Responsiveness and functional attributes of electrically localized terminals of cutaneous C-fibers *in vivo* and *in vitro*. Journal of neurophysiology.

[22] Kwan KY, Glazer JM, Corey DP, Rice FL, Stucky CL (2009). TRPA1 modulates mechanotransduction in cutaneous sensory neurons. The Journal of neuroscience: the official journal of the Society for Neuroscience.

[23] Levy D, Strassman AM (2002). Mechanical Response Properties of A and C Primary Afferent Neurons Innervating the Rat Intracranial Dura. Journal of neurophysiology.

[24] Ferrell BR (1995). The impact of pain on quality of life. A decade of research. The Nursing clinics of North America.

[25] Reyes-Gibby CC (2014). Survival patterns in squamous cell carcinoma of the head and neck: pain as an independent prognostic factor for survival. The journal of pain: official journal of the American Pain Society.

[26] Oliveira KG (2014). Influence of pain severity on the quality of life in patients with head and neck cancer before antineoplastic therapy. BMC Cancer.

[27] Grond S, Zech D, Diefenbach C, Radbruch L, Lehmann KA (1996). Assessment of cancer pain: a prospective evaluation in 2266 cancer patients referred to a pain service. Pain.

[28] Xu MDJ, Brennan MDPD (2010). Timothy J. Guarding Pain and Spontaneous Activity of Nociceptors after Skin versus Skin Plus Deep Tissue Incision. Anesthesiology.

[29] Zhao FY (2014). *In vivo* electrophysiological recording techniques for the study of neuropathic pain in rodent models. Curr Protoc Pharmacol.

[30] Banik RK, Brennan TJ (2004). Spontaneous discharge and increased heat sensitivity of rat C-fiber nociceptors are present *in vitro* after plantar incision. Pain.

[31] Cain DM, Wacnik PW, Simone DA (2001). Animal models of cancer pain may reveal novel approaches to palliative care. Pain.

[32] Pickering V, Jay Gupta R, Quang P, Jordan RC, Schmidt BL (2008). Effect of peripheral endothelin-1 concentration on carcinoma-induced pain in mice. European journal of pain.

[33] Arcourt A (2017). Touch Receptor-Derived Sensory Information Alleviates Acute Pain Signaling and Fine-Tunes Nociceptive Reflex Coordination. Neuron.

[34] Page AJ (2004). The ion channel ASIC1 contributes to visceral but not cutaneous mechanoreceptor function. Gastroenterology.

[35] Holzer P (2015). Acid-Sensing Ion Channels in Gastrointestinal Function. Neuropharmacology.

[36] Peiris M (2017). Peripheral KV7 channels regulate visceral sensory function in mouse and human colon. Molecular Pain.

[37] Lam DK, Schmidt BL (2010). Serine proteases and protease-activated receptor 2-dependent allodynia: a novel cancer pain pathway. Pain.

[38] Prato V (2017). Functional and Molecular Characterization of Mechanoinsensitive “Silent” Nociceptors. Cell reports.

[39] Wu Ping, Max Grayson DA, Hung Chia-Nung, Ruparel Shivani (2018). Characterization of Sensory Neuronal Subtypes Innervating Mouse Tongue. PLoS One.

[40] Seal RP (2009). Injury-induced mechanical hypersensitivity requires C-low threshold mechanoreceptors. Nature.

[41] Usoskin D (2015). Unbiased classification of sensory neuron types by large-scale single-cell RNA sequencing. Nature neuroscience.

[42] Abraira, V. E. & Ginty, D. D. The Sensory Neurons of Touch. *Neuron* **79**, 10.1016/j.neuron.2013.07.051 (2013). 10.1016/j.neuron.2013.07.051 PMC3811145 23972592

[43] Christophe D, Ibtissam B-W, Bernard D (1994). Innervation of Putative Rapidly Adapting Mechanoreceptors by Calbindin- and Calretinin-immunoreactive Primary Sensory Neurons in the Rat. European Journal of Neuroscience.

[44] Li L (2011). The functional organization of cutaneous low-threshold mechanosensory neurons. Cell.

[45] Biggs JE (2008). Effect of SB-750364, a specific TRPV1 receptor antagonist, on injury-induced ectopic discharge in the lingual nerve. Neurosci Lett.

[46] Bryant BP, Moore PA (1995). Factors affecting the sensitivity of the lingual trigeminal nerve to acids. Am J Physiol.

[47] Pittman DW, Contreras RJ (1998). Responses of single lingual nerve fibers to thermal and chemical stimulation. Brain Res.

